# Synergistic Effects of Photo-Irradiation and Curcumin-Chitosan/Alginate Nanoparticles on Tumor Necrosis Factor-Alpha-Induced Psoriasis-Like Proliferation of Keratinocytes

**DOI:** 10.3390/molecules24071388

**Published:** 2019-04-09

**Authors:** Clinton Gomez, Chawanphat Muangnoi, Feaungthit Niyamissara Sorasitthiyanukarn, Jongkonnee Wongpiyabovorn, Pornchai Rojsitthisak, Pranee Rojsitthisak

**Affiliations:** 1 Biomedicinal Chemistry Program, Department of Biochemistry and Microbiology, Faculty of Pharmaceutical Sciences, Chulalongkorn University, Bangkok 10330, Thailand; cbgomez@up.edu.ph; 2 Natural Products for Ageing and Chronic Diseases Research Unit, Chulalongkorn University, Bangkok 10330, Thailand; chawanphat.mua@gmail.com (C.M.); Feuangthit.N@chula.ac.th (F.N.S.); pornchai.r@chula.ac.th (P.R.); 3 Institute of Nutrition, Mahidol University, Nakhon Pathom 73170, Thailand; 4 Metallurgy and Materials Science Research Institute, Chulalongkorn University, Bangkok 10330, Thailand; 5 Center of Excellence in Immunology and Immune Mediated Diseases, Division of Immunology, Department of Microbiology, Faculty of Medicine, Chulalongkorn University, Bangkok 10330, Thailand; jongkonneew@gmail.com; 6 Department of Food and Pharmaceutical Chemistry, Faculty of Pharmaceutical Sciences, Chulalongkorn University, Bangkok 10330 Thailand

**Keywords:** photo-irradiation, curcumin, chitosan/alginate nanoparticles, psoriasis, LED light

## Abstract

Psoriasis is a chronic inflammatory skin disease characterized by hyperproliferation of the epidermal cells and is clinically presented as thick, bright red to pink plaques with a silvery scale. Photodynamic therapy (PDT) using visible light has become of increasing interest in the treatment of inflammatory skin diseases. In this study, we demonstrate that a combination of curcumin-loaded chitosan/alginate nanoparticles (Cur-CS/Alg NPs) and blue light emitting diodes (LED) light irradiation effectively suppressed the hyperproliferation of tumor necrosis factor-alpha (TNF-α)-induced cultured human kerlatinocyte (HaCaT) cells. The Cur-CS/Alg NPs were fabricated by emulsification of curcumin in aqueous sodium alginate solution and ionotropic gelation with calcium chloride and chitosan using an optimized formulation derived from a Box-Behnken design. The fabricated Cur-CS/Alg NPs were characterized for their particle size, zeta potential, encapsulation efficiency, and loading capacity. The surrogate 3-(4,5-dimethylthiazol-2-yl)-2,5-diphenyl tetrazolium bromide (MTT) assay, to measure the relative number of viable cells, showed that the CS/Alg NPs were nontoxic to normal HaCaT cells, while 0.05 µg/mL and 0.1 µg/mL of free curcumin and Cur-CS/Alg NPs inhibited the hyperproliferation of HaCaT cells induced by TNF-α. However, the Cur-CS/Alg NPs demonstrated a stronger effect than the free curcumin, especially when combined with blue light irradiation (10 J/cm^2^) from an LED-based illumination device. Therefore, the Cur-CS/Alg NPs with blue LED light could be potentially developed into an effective PDT system for the treatment of psoriasis.

## 1. Introduction

Psoriasis is a chronic inflammatory skin disease that affects at least 100 million individuals worldwide, with a prevalence in countries that ranges from 0.09% to 11.43% [1]. The most common form of the disease, chronic plaque type psoriasis or psoriasis vulgaris, accounts for about 90% of all psoriasis cases. Some of the main features of psoriasis include keratinocyte hyperproliferation, incomplete differentiation, and inflammation, which presents clinically in patients as erythematous skin lesions covered by thick silvery scales [2,3]. Typical sites of psoriatic plaques include the scalp, extensor surfaces of the elbows, anterior aspects of the lower legs, umbilicus, and natal cleft [4]. The severity of psoriasis ranges from mild, with a limited number of localized inflammatory skin lesions, to a more severe state involving widespread plaques that cover more than 10% of the body surface area [5]. The complete etiology and pathophysiology of psoriasis remains unclear. There is still no definitive cure for psoriasis and treatment options are limited to the control of the disease symptoms. Management of psoriatic symptoms depends on the severity of the disease. Topical therapies are used to treat mild forms of psoriasis, while phototherapy and systemic therapy are reserved for moderate to severe cases of psoriasis [6].

The American Academy of Dermatology considers phototherapy using ultraviolet (UV) light as the mainstay treatment for moderate to severe psoriasis. Phototherapy with UV light is usually available as ultraviolet B (UVB) or ultraviolet A (UVA) light alone or in combination with substances that can be activated by these wavelengths, such as in the case of psolaren plus UVA. Although generally effective, decreased patient compliance to UV phototherapy has been reported, which is mainly due to the increased risk of skin cancer associated with UV light exposure and a painful burning itch that persists at the treatment area for months after UV phototherapy [7]. Thus, in recent years, there has been a growing interest in the use of visible light for the treatment of inflammatory skin diseases. Specifically, blue light (wavelength range from 400 to 480 nm) has been shown to have fewer harmful side effects to mammalian cells than UV irradiation [8]. Furthermore, blue light irradiation has been observed to regulate the proliferation and differentiation of human skin cells [9].

Photodynamic therapy (PDT) refers to a specific type of phototherapy that involves the three components of visible light, a photosensitizer, and oxygen [10]. In a PDT system, the photosensitizer is administered and taken up by the target cells and then a measured dose of visible light of the appropriate wavelength is used to irradiate the target site. Depending on the dose of the photosensitizer and light used in the system, PDT may inhibit cell proliferation and inflammatory responses [11,12]. The development of a PDT system that can be optimized to produce the desired effects on keratinocyte proliferation would, therefore, prove potentially advantageous in the management of psoriasis. Therefore, this study was under taken to develop a PDT system optimized for the treatment of psoriasis using curcumin-loaded chitosan/alginate nanoparticles (Cur-CS/Alg NPs) as photosensitizers and blue light emitting diodes (LED) as the light source.

Curcumin is a bioactive phytochemical extracted from the roots of turmeric (*Curcuma longa* L.), and has been proven safe for clinical use and reported to exhibit several pharmacological properties [13]. With a maximum light absorption peak at 415 to 430 nm, curcumin could be a promising photosensitizer for use in blue light-based PDT. However, this potential application of curcumin is hampered by its low solubility, poor stability, and extensive metabolism [14]. Previous studies have suggested two possible solutions. The first is the synthesis of curcumin derivatives, such as curcumin diethyl disuccinate and curcumin diglutaric acid [15,16,17], which possess improved physicochemical properties and pharmacological effects. The second is the encapsulation in CS/Alg NPs, which is a biocompatible drug delivery system that could improve the efficacy of curcumin [18,19,20,21,22]. The interest of this study was on the investigation of a third approach, which involves amplification of the effects of curcumin through the application of a combination of CS/Alg nano-encapsulated curcumin and blue LED light irradiation.

## 2. Results and Discussion

### 2.1. Optimization of the Cur-CS/Alg NP Formulation

The use of response surface methodology (RSM) is an effective approach in the optimization of the Cur-CS/Alg NP formulation [20,22]. In this study, a Box-Behnken statistical design (BBD) was employed to identify the optimum curcumin concentrations (*X*_1_) and Tween^®^ 80 (*X*_2_) and the CS/Alg mass ratio (*X*_3_) that would result in a Cur-CS/Alg NP formulation with a particle size and zeta potential within the desirable range and a maximal encapsulation efficiency (EE) and loading capacity (LC). The observed responses of the Cur-CS/Alg NP formulation at different levels of the factors are shown in Table 1, where the three dependent values ranged from 199 to 1120 nm for particle size (*Y*_1_), −30.8 mV to −10.8 mV for zeta potential (*Y*_2_), 45.7% to 54.5% for the EE (*Y*_3_), and 4.0% to 27.4% for the LC (*Y*_4_).

Regression analysis was performed using the Design Expert^®^ software to simultaneously fit the responses obtained from all 15 formulations into linear, interaction, and quadratic models. Table 2 summarizes the sequential model sum of squares, lack of fit, and model summary statistics obtained from the regression analysis.

The most suitable model for each response was identified from having an adjusted R^2^ and predicted R^2^ close to 1, with a significant sequential model sum of squares (*p* < 0.05) and non-significant lack of fit (*p* > 0.05). Thus, the particle size, zeta potential, and EE were fitted to a quadratic second-order polynomial model. In case of LC, although the adjusted R^2^ and predicted R^2^ values of the linear model were approx. 0.77 or 0.65, respectively, they were the highest values compared to those of the interaction and quadratic models. Consequently, the linear model was generated and interpreted as the best model for this response. The equations for the four generated models are shown in Equations (1)–(4):

*Y*_1_ = 364.77 + 13.92*X*_1_ + 18.06*X*_2_ + 374.18*X*_3_ − 8.80*X*_1_*X*_2_ + 42.05*X*_1_*X*_3_ + 30.34*X*_2_*X*_3_ + 57.02*X*_1_^2^ − 33.47*X*_2_^2^ + 257.95*X*_3_^2^ (1)

*Y*_2_ = −25.77 − 0.0234*X*_1_ + 1.49*X*_2_ − 7.53*X*_3_ + 0.8270*X*_1_*X*_2_ − 0.3109*X*_1_*X*_3_ + 1.34*X*_2_*X*_3_ + 1.21*X*_1_^2^ − 0.8977*X*_2_^2^ + 5.68*X*_3_^2^ (2)

*Y*_3_ = 48.04 + 1.08*X*_1_ − 0.2771*X*_2_ − 1.47*X*_3_ + 0.1547*X*_1_*X*_2_ − 1.59*X*_1_*X*_3_ − 0.2248*X*_2_*X*_3_ + 0.5616*X*_1_^2^ − 1.67*X*_2_^2^ + 1.50*X*_3_^2^ (3)

*Y*_4_ = 11.35 + 6.59*X*_1_ − 0.9431*X*_2_ − 4.34*X*_3_ (4)

**Figure 1:**
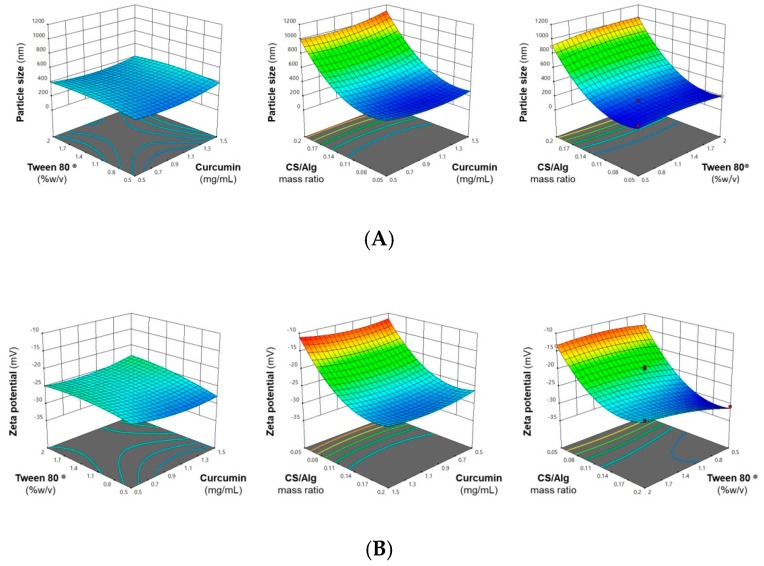

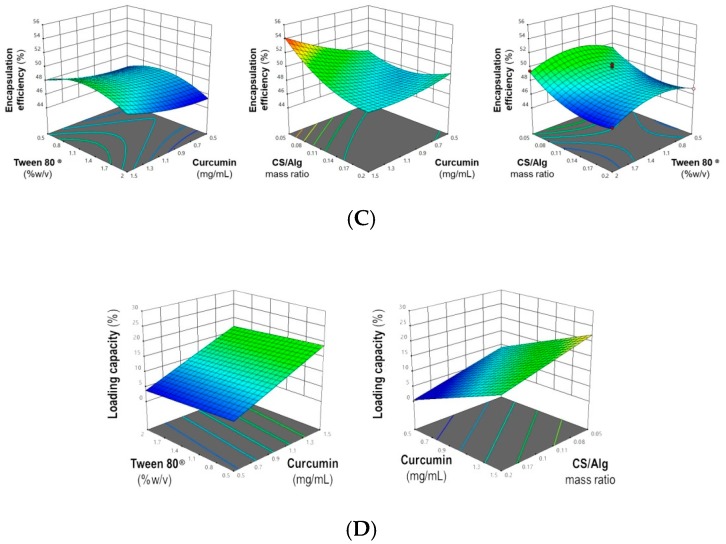
Representative response surface plots showing the effect of the formulation factors (curcumin concentration, Tween^®^ 80 concentration, and CS/Alg mass ratio) on the (**A**) particle size, (**B**) zeta potential, (**C**) encapsulation efficiency (%), and (**D**) loading capacity (%).

In the polynomial equation, a positive value indicates a synergistic effect through which the response increases proportionally to the factor. In contrast, a negative value indicates an antagonistic effect through which the response decreases proportionally to the factor. These synergistic and antagonistic interactions between factors and responses were also confirmed by the three-dimensional response surface plots, shown in Figure 1. Lastly, Table 3 summarizes the significance of each factor studied in the models generated for each response.

The curcumin concentration had a significant synergistic effect on the EE and LC. Fattahpour et al. [23] suggested that the drug concentration was the main driving force in drug entrapment in NPs. On the other hand, the curcumin concentration had no significant effect on the particle size and zeta potential. A possible explanation for this is that the hydrophobic nature of curcumin results in drug loading in the NPs being mainly through physical entrapment since curcumin offers no significant electrostatic interactions with the chitosan (CS) and alginate (Alg) polar groups that could affect the polymer cross linking and surface charge density [24]. 

The Tween^®^ 80 concentration had no significant effect on the particle size, EE, and LC, but based on Equation (2), it had a significant synergistic effect on the zeta potential, where a less negative zeta potential was observed with higher Tween^®^ 80 concentrations. Lertsutthiwong et al. [25] also suggested that being amphiphilic, Tween^®^ 80 can interact with curcumin by its hydrophobic tail and with Alg by its hydrophilic head. It is possible that binding of Tween^®^ 80 with the Alg shell following crosslinking with CaCl_2_ resulted in a reduced surface charge density, leading to a less negative zeta potential.

The CS/Alg mass ratio had a significant effect on the Cur-CS/Alg NP size and a significant antagonistic effect on the zeta potential, EE, and LC. The formation of Cur-CS/Alg NPs involves the spontaneous electrostatic interaction between the negatively charged carboxylate groups on Alg and the positively charged protonated amino groups on CS [26]. The use of a higher CS/Alg mass ratio results in a better formation of the CS network on the surface of the CaCl_2_-Alg shell, leading to the production of larger sized NPs. For this study, a particle size of 200 to 300 nm was desired, since it has been demonstrated that NPs in this size range are capable of penetration, localization, and drug diffusion into the layers of the skin [24]. From the results, it was found that the use of low to medium CS/Alg mass ratios produced NPs within the desired size range. 

The zeta potential is the electrostatic potential near the surface of the particles and is used as an indicator of the stability of NP suspensions [22]. From the results, it was observed that the use of medium to high CS/Alg mass ratios resulted in the production of Cur-CS/Alg NPs with a zeta potential from −20 mV to −30 mV, a range wherein improved stability is imparted to the NPs. The EE and LC of the CS/Alg NPs were relatively low because hydrophilic polymers (CS and Alg) were used to encapsulate a relatively hydrophobic molecule (curcumin). However, a sufficient EE and LC could be obtained even with the use of a low CS/Alg mass ratio, but increasing the CS/Alg mass ratio further decreased the EE and LC.

Using RSM, the optimal formulation was derived as a CS/Alg mass ratio of 0.08:1, curcumin concentration of 1.5 mg/mL, and Tween^®^ 80 concentration of 0.5% (*w*/*v*). To validate this optimal formulation, Cur-CS/Alg NPs were prepared using the optimized conditions and the observed values of the responses were compared with the predicted values. The results in Table 4 show that the observed and predicted values of the responses were acceptable with a low % error, which showed adequate precision for the prediction of the optimized condition. 

### 2.2. Optimization of the Blue LED-Based Illumination Device

The specific blue LED array device was fabricated as outlined in Section 3.5. The irradiance emitted by the LED array decreased with the increasing irradiation distance (light to plate distance) and can be continuously adjusted in the range from 28 to 181 mW/cm^2^. A better homogeneity of the irradiance was found for longer irradiation distances. Since the most commonly used irradiance for in vitro PDT studies is approximately 20 mW/cm^2^ [27], the optimal irradiation distance for the current system was set at 18 cm. In addition, the optimal operation for photodynamic therapy with this illumination device was as follows; 5 min prior to irradiation for machine equilibration, followed by 5 min of photo-irradiation. With this condition, the temperature for each experiment was controlled at 27 to 35 °C. Using the surrogate 3-(4,5-dimethylthiazol-2-yl)-2,5-diphenyl tetrazolium bromide (MTT) assay, we observed no change in the cell viability of keratinocyte after irradiation under this condition. Hence, this protocol provides an appropriate condition to investigate the effects of LED exposure in combination with other compounds (e.g., curcumin or Cur-CS/Alg NPs) in photodynamic therapy in vitro. 

### 2.3. In Vitro Cytotoxicity/Anti-Proliferation Effect of the PDT on Normal and Tumor Necrosis Factor-Alpha (TNF-α)-Induced Cultured Human Kerlatinocyte (HaCaT) Cells

**Figure 2:**
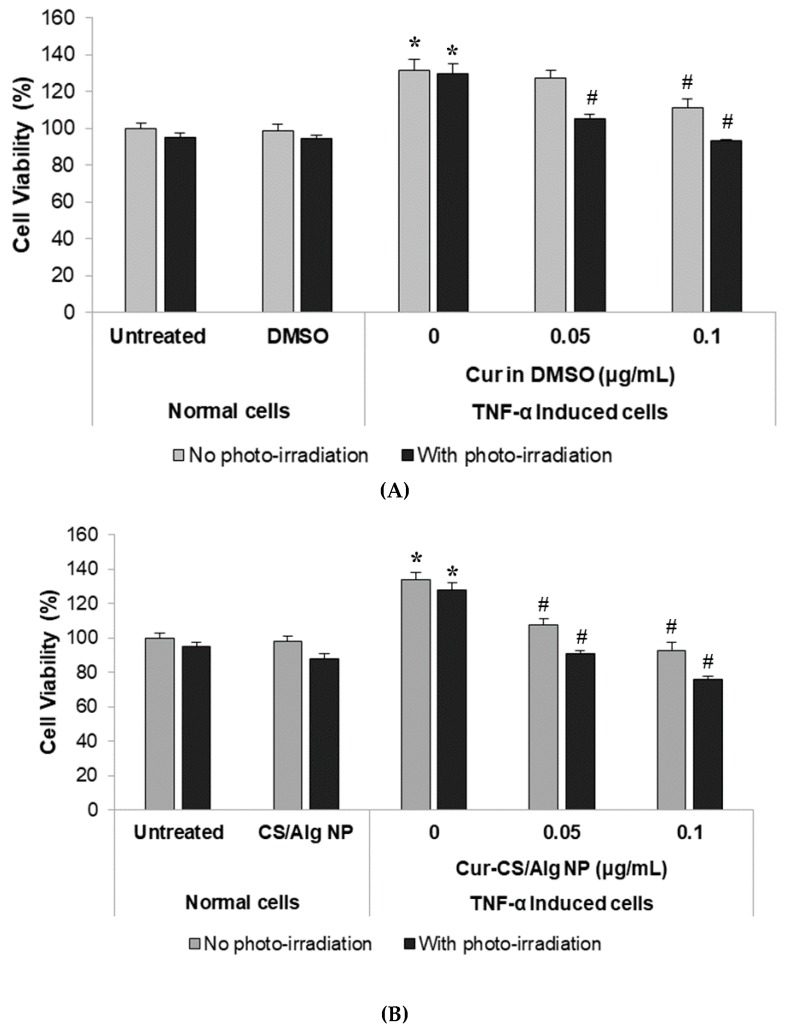
Induction of psoriasis-like proliferation of HaCaT cells by TNF-α and inhibition of the TNF-α-induced cell proliferation after treatment with (**A**) curcumin in DMSO and (**B**) Cur-CS/Alg NPs at 0.05 µg/mL and 0.1 µg/mL with and without photo-irradiation (10 J/cm^2^). Controls for free curcumin and Cur-CS/Alg NPs were DMSO and CS/Alg NPs, respectively. Data are presented as the mean (± SD) relative number of viable cells compared to the untreated control group (100%), and are derived from four replications. * *p* < 0.05 indicates significant differences from the untreated normal cells, ^#^ *p* < 0.05 indicates significant difference from the TNF-α-induced cells treated with the control (DMSO or empty CS/Alg NPs).

The results of the MTT assay to evaluate the cytotoxic/anti-proliferative effects of photo-irradiation in combination with free curcumin dissolved in DMSO or loaded in CS/Alg NPs are shown in Figure 2. The stimulation of HaCaT cells with TNF-α resulted in an increased number of viable cells to ca. 135% relative to the unstimulated cells, confirming the responsiveness of HaCaT cells to TNF-α. The dimethyl sulfoxide (DMSO) and CS/Alg NPs controls (for free curcumin and Cur-CS/Alg NPs, respectively) showed no significant difference in the number of viable cells compared to the respective untreated or TNF-α-stimulated HaCaT cells, and so DMSO and CS/Alg NPs at 0.5% (*v*/*v*) have no significant cytotoxicity to HaCaT cells or inhibition of the TNF-α-induced proliferation. 

In accord, our previous work demonstrated that empty CS/Alg NPs at 0.1 to 0.5% (*v*/*v*) were not cytotoxic to HaCaT cells (Gomez et al., unpublished data), while higher concentrations of CS/Alg NPs at 1.0% (*v*/*v*) were mildly toxic to the cells [28]. Furthermore, the data established the non-destructive potential of photo-irradiation from the blue LED device fabricated in this study, where the exposure of normal HaCaT cells to the blue light (425 nm) at 20 mW/cm^2^ for 5 min (equivalent to a light dose of about 10 J/cm^2^) did not result in any significant cytotoxic effects. This observation is consistent with a previous study that reported that LED irradiation with 412 to 426 nm blue light was toxic to keratinocytes only at higher light doses (33–100 J/cm^2^) [9].

In order to simulate a condition of hyperproliferative keratinocytes similar to that observed in psoriasis, HaCaT cells were treated with TNF-α for 24 h. The induction with TNF-α successfully increased the relative number of viable cells to 135.5% of that of the control group (Figure 2). Upon treatment with free curcumin at 0.05 µg/mL and 0.1 µg/mL, the relative number of viable TNF-α-induced cells decreased by 8.6% and 24.4%, respectively, (Figure 2A). With subsequent photo-irradiation of the free curcumin-treated cells, the viable cell number was further decreased by 30.8% and 42.7%, respectively (Figure 2A). On the other hand, treatment of the TNF-α-induced cells with Cur-CS/Alg NPs decreased the number of viable cells by about 26.0% and 41.0% at equivalent curcumin concentrations of 0.05 µg/mL and 0.1 µg/mL, respectively (Figure 2B). The loss in viable cell numbers of the Cur-CS/Alg NP treated cells was increased to 42.6% and 57.7%, respectively, after exposure to the blue LED light (Figure 2B).

The desired effect of PDT is the reduction of the keratinocyte (here HaCaT cells) proliferation caused by TNF-α without decreasing the cell viability below that of untreated cells. The treatments that produced this desired effect were 0.1 µg/mL of free curcumin and 0.05 µg/mL of Cur-CS/Alg NPs followed by subsequent exposure to 10 J/cm^2^ of blue LED light. Treatment with 0.1 µg/mL of free curcumin or 0.05 µg/mL of Cur-CS/Alg NPs followed by photo-irradiation resulted in a decreased number of viable cells to 93.0% and 90.6%, respectively, which are significantly lower than that of the TNF-α-induced HaCaT cells, but not significantly different from the cell viability of the untreated normal HaCaT cells. 

These results suggest that Cur-CS/Alg NPs are more effective in reducing the cell proliferation induced by TNF-α than free curcumin, which might be due to the enhanced cellular uptake of the Cur-CS/Alg NPs compared to free curcumin. For example, it was previously demonstrated that encapsulation of hydrophobic molecules, like curcumin and its derivatives, may increase the drug effectiveness by improvement of its delivery into the cell [18,19,20,22]. The results also demonstrated that a further reduction in the number of viable cells was obtained when the treatment was combined with photo-irradiation. Specifically, the reduced HaCaT proliferation was more noticeable for the combination of blue LED light irradiation in the presence of Cur-CS/Alg NPs than in the presence of free curcumin. A possible explanation for the increased efficacy of Cur-CS/Alg NPs is the photoprotection provided to the encapsulated curcumin, and the low-level sustained release of curcumin from the Cur-CS/Alg NPs, as supported by a previous study [29]. This means that curcumin is protected from premature photodegradation during delivery and only the released curcumin inside the cells interacts with the blue light for an increased effect. Therefore, in summary, these effects are synergistic and the combination of Cur-CS/Alg NPs and photo-irradiation results in a higher loss of TNF-α-induced proliferation, at least in HaCaT cells. This observation suggests a potential approach in PDT for the PDT of psoriasis.

### 2.4. Physical Stability

**Figure 3:**
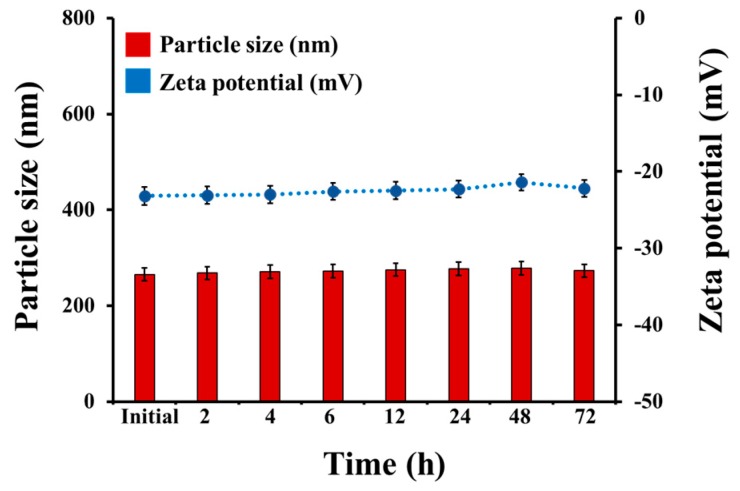
Physical stability of Cur-CS/Alg NPs in cell culture medium (pH 7.4) (*n* = 3).

The physical stability of the optimized Cur-CS/Alg NPs in culture medium (CM) was evaluated at 37 °C for 72 h. The size and the zeta potential of Cur-CS/Alg NPs in CM were unchanged during incubation (Figure 3). In other words, there was no dissociation of Cur-CS/Alg NPs into small fragments or agglomeration of Cur-CS/Alg NPs, suggesting that no digestion of NPs into smaller particles occurred during the sample treatment of TNF-α-induced psoriasis-like proliferation of HaCaT cells.

### 2.5. In Vitro Cellular Uptake

**Figure 4:**
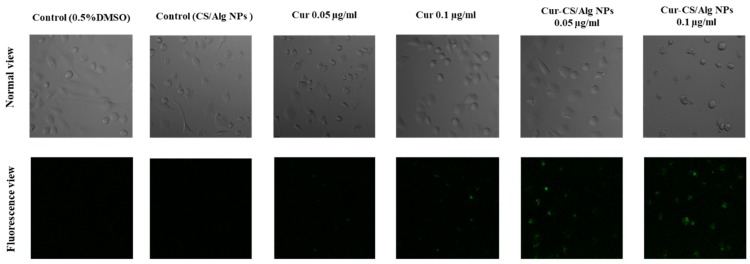
Cellular uptake of curcumin and Cur-CS/Alg NPs viewed by fluorescence microscopy in HaCaT cells.

The cellular uptake of curcumin and Cur-CS/Alg NPs was investigated in HaCaT cells. Fluorescence microscopy images of each treatment are shown in Figure 4. Curcumin and Cur-CS/Alg NPs emitted fluorescent light under a fluorescence microscope, whereas the control cells treated with DMSO or empty nanoparticles had no fluorescent signal. Interestingly, the Cur-CS/Alg NPs-treated groups had higher fluorescent intensity than curcumin at the equivalent concentration of curcumin. These results indicated that the Cur-CS/Alg NPs improved the cellular uptake of curcumin.

## 3. Materials and Methods

### 3.1. Materials

CS, with a molecular weight of 22,000 Da and degree of deacetylation of 90%, was donated by Marine Bio Resources Co., Ltd., Samut Sakorn, Thailand. Sodium alginate with a medium viscosity was purchased from Sigma, St Louis, MO, USA. Curcumin was prepared and characterized from the previously published method [16]. Non-ionic surfactant named Tween^®^ 80 (or Polysorbate 80) was supplied from Acros Organics, Thermo Fisher Scientific, Morris Plains, NJ, USA. All other chemicals were of analytical grade.

### 3.2. Preparation of the Cur-CS/Alg NPs

Na-Alg and CaCl_2_ solutions were dissolved in ultrapure water, while CS was dissolved in 1% (*v*/*v*) acetic solution. The pH of the Alg and CS solutions were adjusted to 4.9 and 4.6, respectively, using 1% (*v*/*v*) acetic acid and 4% (*w*/*v*) NaOH, respectively. Tween^®^ 80 was used to emulsify the ethanolic curcumin and Alg solution through micelle formation. The Cur-CS/Alg NP formulations were prepared by oil in water (o/w) emulsification followed by ionotropic gelation using a modified version of the described methods [20,22]. Briefly, 1 mL of ethanolic curcumin solution at various concentrations was added dropwise into 20 mL of Alg solution (0.6 mg/mL) containing various concentrations of Tween^®^ 80 under vigorous stirring (1000 rpm) for 10 min. After sonication for 15 min, 4 mL of CaCl_2_ solution (0.67 mg/mL) was added dropwise into the resulting o/w emulsion and stirred continuously for 30 min, before addition of 4 mL of the CS solution. After being continuously stirred for 30 min, the resulting Cur-CS/Alg NP suspension was equilibrated overnight before characterization. The empty (unloaded) CS/Alg NPs were made in the same manner only without the addition of the curcumin.

### 3.3. Characterization of the Cur-CS/Alg NPs

The particle size and zeta potential were determined using dynamic light scattering with a Nano-ZS Zeta-sizer (Malvern Instruments, Worcestershire, UK). The encapsulation efficiency (EE) and loading capacity (LC) of the CS/Alg NPs with curcumin were determined using an indirect method as previously described [22]. In brief, the Cur-CS/Alg NP suspension was separated from the aqueous media by ultracentrifugation at 105,000× *g* at 25 °C for 45 min. After freeze-drying at −85 °C for 12 h, the dry mass of the lyophilized NPs was recorded. Meanwhile, the amount of curcumin in the supernatant was determined using UV-vis spectroscopy (Agilent Cary 60, Agilent Technology Ltd., Santa Clara, CA, USA) to measure the absorbance at 425 nm. The EE (%) and LC (%) of the CS/Alg NPs were then calculated using Equations (5) and (6), respectively:

EE (%) = 100 × (Cur in formulation − Cur in supernatant)/(Cur in formulation)(5)

LC (%) = 100 × (Cur in formulation − Cur in supernatant)/(dry mass of NPs)(6)

### 3.4. Design and Optimization of the Cur-CS/Alg NP Formulation

The Cur-CS/Alg NP formulation was designed and optimized using BBD and RSM, as provided by the Design-Expert^®^ software (Trial-Version10.0.0, Stat Ease, MN, USA). The BBD was applied to determine the optimal level of factors (independent variables) that give responses (dependent variables). The independent variables were the curcumin concentration (*X*_1_), Tween^®^ 80 concentration (*X*_2_), and CS/Alg mass ratio (*X*_3_). The dependent variables were the particle size (*Y*_1_), zeta potential (*Y*_2_), EE (*Y*_3_), and LC (*Y*_4_). The ranges of the independent variables and the constraints for the dependent variables are summarized in Table 5.

### 3.5. Fabrication of the Blue LED Based Illumination Device

**Figure 5:**
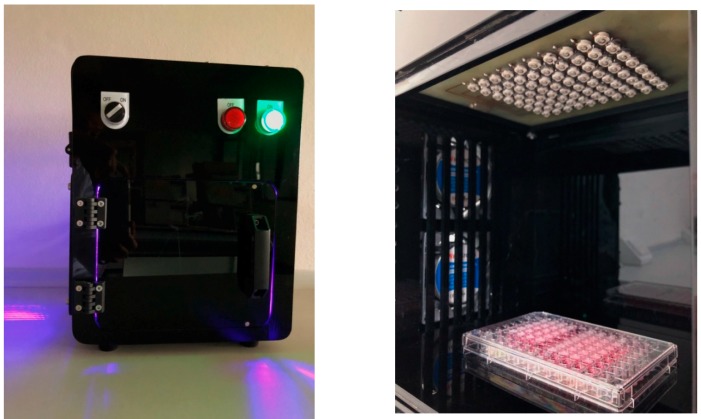
Photograph of the illumination device (**left**) and the 10 × 8 blue LED array (**right**).

An LED based illumination device (Figure 5) specifically intended for the in vitro evaluation of the photodynamic effect of the Cur-CS/Alg NPs was developed based on a previous study [27]. The device included a blue LED array composed of 80 LEDs centered at 425 nm, a cooling fan system to prevent overheat, and a specific-designed case. The illumination area was designed to be able to accommodate the standard 96-well plates used in cell-based assays. A movable platform installed in the device allowed the distance between the LED array and the illuminated area (the tissue culture plate) to be adjusted from 0 to 18 cm in increments of 4.5 cm. To determine the effect of the distance on the irradiance emitted by the LED array, the irradiances were measured at nine spots on the illumination area for each increment of distance.

### 3.6. Cell Culture

The immortalized HaCaT human keratinocyte line was obtained from American Type Culture Collection (Manassas, VA). The cells were cultured in CM, comprised of Dulbecco’s modified Eagle’s medium (DMEM) supplemented with 10% (*v*/*v*) heat-inactivated fetal bovine serum and 1% (*v*/*v*) penicillin-streptomycin at 37 °C in a humidified atmosphere of 5% CO_2_/95% air.

### 3.7. Induction of Proliferation of HaCaT Cells by TNF-α

The HaCaT cells in CM were seeded in 96-well plates at a density of 5 × 10^3^ cells per well and incubated at 37 °C in a humidified atmosphere of 5% CO_2_/95% air for 24 h. The cultured cells were then washed with serum free CM and TNF-α in serum free CM was added to 10 ng/mL and the cells were incubated for 24 h.

### 3.8. Cell Treatment and Photo-Irradiation

Control and TNF-α-induced HaCaT cells were used to investigate the individual and combined effects of curcumin, Cur-CS/Alg NPs, and blue LED photo-irradiation on keratinocyte proliferation. Free curcumin dissolved in DMSO and Cur-CS/Alg NPs were added to the cells at final curcumin concentrations of 0.05 and 0.1 µg/mL, while DMSO and CS/Alg NPs at 0.5% (*v*/*v*) and 1% (*v*/*v*), respectively, were used as controls. The treated cells were incubated for 1 h and then the plate was exposed to the blue LED light for 5 min. A control plate not subjected to photo-irradiation was also prepared. The number of viable cells after treatment and photo-irradiation was then assessed by the MTT assay to determine the number of viable cells relative to that for the untreated control culture (Section 3.9).

### 3.9. Cell Viability Assay

The MTT assay is based on the ability of mitochondrial dehydrogenase enzymes in living cells to convert the yellow water-soluble tetrazolium salt MTT into dark blue formazan crystals. The cells were washed and incubated in serum-free CM containing 0.5 mg/mL of MTT solution at 37 °C in a humidified atmosphere of 5% CO_2_/95% air for 4 h. The media was removed and 200 µL of DMSO was then added to each well to dissolve the formazan crystals. The absorbance of formazan was measured at 540 nm by a microplate reader (CLARIOstar, BMG Labtech, Ortenberg, Germany). Four replicates of each experiment were performed.

### 3.10. Physical Stability in Cell Culture Medium

The physical stability of the Cur-CS/Alg NPs was determined in CM using the modified version of a previous protocol [22]. Briefly, 1 mL of freshly prepared Cur-CS/Alg NP suspension from the optimal formulation was dispersed in 9 mL of CM and incubated at 37 °C for 72 h. At specific time intervals, the particle size and zeta potential of the Cur-CS/Alg NPs were measured as previously described.

### 3.11. In Vitro Cellular Uptake

HaCaT cells were seeded at a density of 5 × 10^3^ cells per well and incubated at 37 °C in a humidified atmosphere of 5% CO_2_/95% air for 24 h. The cells were washed with serum-free CM and treated with curcumin or Cur-CS/Alg NPs at final Cur concentrations of 0.05 and 0.1 µg/mL. DMSO at 0.5% *v/v* and CS/Alg NPs at 1% *v/v* were used as controls. After incubation for 4 h, the cells were washed with PBS and subsequently imaged under a fluorescence microscope (Nikon, ECLIPSE TiU, Watertown, MA, USA) using a filter excitation and emission at 400 and 470 nm, respectively. Images were processed using Nikon software.

### 3.12. Statistical Analysis

All numerical data in this study are presented as the mean ± standard deviation (SD). Statistical analysis was performed by one-way analysis of variance (ANOVA) followed by Tukey’s post hoc comparison, accepting significance at the *p* < 0.05 level.

## 4. Conclusions

This study successfully optimized the components of the desired PDT system, a combination of Cur-CS/Alg NPs and blue LED light, for the treatment of TNF-α-induced psoriasis-like proliferation of keratinocytes. The optimal formulation of the Cur-CS/Alg NPs was 1.5 mg/mL curcumin, 0.5% (*w*/*v*) Tween^®^ 80, and a CS/Alg mass ratio of 0.08:1, while the optimal conditions of the blue LED device were a blue light irradiation of 10 J/cm^2^ for 5 min at a distance of 18 cm from the LED array to the illumination area. In general, the administration of Cur-CS/Alg NPs with blue LED light could be potentially developed into an effective PDT system for the treatment of psoriasis. Therefore, further investigation of the anti-psoriasis properties of Cur-Cs/Alg NPs with blue LED light in animal models based on the in vitro results presented here is recommended.

## Figures and Tables

**Table 1:** Factors and observed responses in the Box-Behnken statistical design for optimization of the Cur-CS/Alg NP formulation.

Run	Factor			Response			
	*X*_1_ (mg/mL)	*X*_2_ (% *w*/*v*)	*X*_3_	*Y*_1_ (nm)	*Y*_2_ (mV)	*Y*_3_ (%)	*Y*_4_ (%)
1	0.5	0.5	0.1:1	282 ± 63	−22.3 ± 5.7	46.9 ± 0.2	7.1 ± 1.6
2	1.5	0.5	0.1:1	263 ± 57	−24.3 ± 1.8	49.0 ± 0.6	24.0 ± 1.3
3	0.5	2	0.1:1	330 ± 14	−22.1 ± 4.4	45.7 ± 0.2	4.7 ± 1.8
4	1.5	2	0.1:1	294 ± 46	−20.6 ± 2.2	48.8 ± 0.3	14.6 ± 1.0
5	0.5	1	0.05:1	295 ± 61	−11.4 ± 2.5	48.4 ± 0.2	9.9 ± 5.9
6	1.5	1	0.05:1	281 ± 34	−10.8 ± 5.2	54.5 ± 1.6	27.4 ± 9.6
7	0.5	1	0.2:1	984 ± 80	−27.4 ± 1.4	48.9 ± 0.1	4.0 ± 0.7
8	1.5	1	0.2:1	1120 ±8 4	−28.4 ± 1.6	48.2 ± 0.4	12.5 ± 2.9
9	1	0.5	0.05:1	267 ± 52	−14.3 ± 1.1	49.2 ± 0.8	10.9 ± 3.7
10	1	2	0.05:1	199 ± 37	−13.5 ± 6.0	49.5 ± 1.5	18.1 ± 4.1
11	1	0.5	0.2:1	899 ± 6	−30.8 ± 1.4	46.9 ± 0.6	7.6 ± 3.4
12	1	2	0.2:1	992 ± 180	−25.4 ± 1.6	46.0 ± 0.2	6.0 ± 2.2
13	1	1	0.1:1	255 ± 88	−25.0 ± 2.2	48.8 ± 0.3	11.9 ± 5.1
14	1	1	0.1:1	245 ± 102	−22.4 ± 1.0	49.2 ± 0.9	12.3 ± 5.4
15	1	1	0.1:1	287 ± 37	−21.8 ± 0.8	47.8 ± 1.1	11.8 ± 4.3

**Table 2:** Summary of the results of the regression analysis of the responses.

Response	Model	Sequential *p*-Value	Lack of Fit *p*-Value	Adjusted R^2^	Predicted R^2^	Remark
*Y*_1_ (Particle size)	Linear	<0.0001	0.0198	0.8251	0.7453	
	2FI	0.9273	0.0140	0.7723	0.4327	
	Quadratic	0.0004	0.2076	0.9882	0.9236	Suggested
*Y*_2_ (Zeta potential)	Linear	0.0005	0.2065	0.7263	0.5988	
	2FI	0.8404	0.1569	0.6591	0.1750	
	Quadratic	0.0030	0.8698	0.9594	0.9002	Suggested
*Y*_3_ (EE)	Linear	0.0520	0.1363	0.3517	−0.0626	
	2FI	0.2574	0.1471	0.4471	−0.7347	
	Quadratic	0.0082	0.6047	0.9010	0.6598	Suggested
*Y*_4_ (LC)	Linear	0.0002	0.0063	0.7726	0.6473	Suggested
	2FI	0.3127	0.0064	0.7948	0.4137	
	Quadratic	0.6684	0.0042	0.7534	−0.9670	

**Table 3:** Significance (*p* < 0.05) of the factors in the generated models.

Factors	*Y*_1_ (Particle size)	*Y*_2_ Zeta potential	*Y*_3_ (EE)	*Y*_4_ (LC)
*X*_1_ (Curcumin concentration)	0.3544	0.9619	0.0062	0.0001
*X*_2_ (Tween^®^ 80 concentration)	0.2315	0.0218	0.2860	0.4089
*X*_3_ (CS/Alg mass ratio)	<0.0001	<0.0001	0.0015	0.0023

**Table 4:** Comparison of the observed and predicted responses of the optimized Cur-CS/Alg NP formulation.

Optimized Formulation (*X*_1_, *X*_2_, *X*_3_)	Response	Predicted Value	Observed Value	Error *
15 mg/mL, 0.5% (*w*/*v*), 0.08:1	*Y*_1_ (nm)	254	245 ± 11	3.6
	*Y*_2_ (mV)	−20.2	−21.1 ± 1.2	4.2
	*Y*_3_ (%)	50.4	47.6 ± 1.8	5.5
	*Y*_4_ (%)	21.5	22.8 ± 0.5	6.1
* Error was calculated as $\frac{\left|\text{observed value}-\text{predicted value}\right|}{\text{predicted value}}\times 100$				

**Table 5:** Variables and responses with their levels and constraints.

	Level			
	Low	Medium	High	
Independent variables (factors)				
*X*_1_ = Curcumin concentration (mg/mL)	0.5	1	1.5	
*X*_2_ = Tween^®^ 80 concentration (% *w*/*v*)	0.5	1	2	
*X*_3_ = CS/Alg mass ratio	0.05:1	0.1:1	0.2:1	
Dependent variables (responses)			**Constraints**	
*Y*_1_ = Particle size (nm)			200–300 nm	
*Y*_2_ = Zeta potential (mV)			−30 mV to −20 mV	
*Y*_3_ = EE (%)			Maximize	
*Y*_4_ = LC (%)			Maximize	

## References

[1] (2016). Global Report on Psoriasis 2016.

[2] Baliwag J., Barnes D.H., Johnston A. (2015). Cytokines in psoriasis. Cytokine.

[3] Boehncke W.H., Schön M.P. (2015). Psoriasis. Lancet..

[4] Higgins E. (2017). Psoriasis. Medicine.

[5] Hawkes J.E., Chan T.C., Krueger J.G. (2017). Psoriasis pathogenesis and the development of novel targeted immune therapies. J. Allergy. Clin. Immunol..

[6] Woo Y.R., Cho D.H., Park H.J. (2017). Molecular mechanisms and management of a cutaneous inflammatory disorder: Psoriasis. Int J. Mol. Sci..

[7] Lapolla W., Yentzer B.A., Bagel J., Halvorson C.R., Feldman S.R. (2011). A review of phototherapy protocols for psoriasis treatment. J. Am. Acad. Dermatol..

[8] Niu T., Tian Y., Cai Q., Ren Q., Wei L. (2015). Red light combined with blue light irradiation regulates proliferation and apoptosis in skin keratinocytes in combination with low concentrations of curcumin. PLoS ONE.

[9] Liebmann J., Born M., Kolb-Bachofen V. (2010). Blue-light irradiation regulates proliferation and differentiation in human skin cells. J. Invest. Dermatol..

[10] Jin Y., Zhang X., Zhang B., Kang H., Du L., Li M. (2015). Nanostructures of an amphiphilic zinc phthalocyanine polymer conjugate for photodynamic therapy of psoriasis. Colloids Surf. B Biointerfaces..

[11] Wang X.L., Wang H.W., Yuan K.H., Li F.L., Huang Z. (2011). Combination of photodynamic therapy and immunomodulation for skin diseases--update of clinical aspects. Photochem. Photobiol. Sci..

[12] Larisch P., Verwanger T., Linecker M., Krammer B. (2014). The interrelation between a pro-inflammatory milieu and fluorescence diagnosis or photodynamic therapy of human skin cell lines. Photodiagnosis Photodyn. Ther..

[13] Anand P., Kunnumakkara A.B., Newman R.A., Aggarwal B.B. (2007). Bioavailability of curcumin: Problems and promises. Mol. Pharm..

[14] Ratnatilaka Na Bhuket P., El-Magboub A., Haworth I.S., Rojsitthisak P. (2017). Enhancement of curcumin bioavailability via the prodrug approach: Challenges and prospects. Eur. J. Drug Metab. Pharmacokinet..

[15] Wongsrisakul J., Wichitnithad W., Rojsitthisak P., Towiwat P. (2010). Antinociceptive effects of curcumin diethyl disuccinate in animal models. J. Health Res..

[16] Wichitnithad W., Nimmannit U., Wacharasindhu S., Rojsitthisak P. (2011). Synthesis, characterization and biological evaluation of succinate prodrugs of curcuminoids for colon cancer treatment. Molecules.

[17] Muangnoi C., Jithavech P., Ratnatilaka Na Bhuket P., Supasena W., Wichitnithad W., Towiwat P., Niwattisaiwong N., Haworth I.S., Rojsitthisak P. (2018). A curcumin-diglutaric acid conjugated prodrug with improved water solubility and antinociceptive properties compared to curcumin. Biosci. Biotechnol. Biochem..

[18] Bhunchu S., Rojsitthisak P. (2014). Biopolymeric alginate-chitosan nanoparticles as drug delivery carriers for cancer therapy. Pharmazie.

[19] Bhunchu S., Rojsitthisak P., Rojsitthisak P. (2015). Effects of preparation parameters on the characteristics of chitosan–alginate nanoparticles containing curcumin diethyl disuccinate J. Drug Deliv. Sci. Technol..

[20] Bhunchu S., Muangnoi C., Rojsitthisak P., Rojsitthisak P. (2016). Curcumin diethyl disuccinate encapsulated in chitosan/alginate nanoparticles for improvement of its in vitro cytotoxicity against MDA-MB-231 human breast cancer cells. Pharmazie.

[21] Luckanagul J.A., Pitakchatwong C., Ratnatilaka Na Bhuket P., Muangnoi C., Rojsitthisak P., Chirachanchai S., Wang Q., Rojsitthisak P. (2018). Chitosan-based polymer hybrids for thermo-responsive nanogel delivery of curcumin. Carbohydr. Polym..

[22] Sorasitthiyanukarn F.N., Muangnoi C., Ratnatilaka Na Bhuket P., Rojsitthisak P., Rojsitthisak P. (2018). Chitosan/alginate nanoparticles as a promising approach for oral delivery of curcumin diglutaric acid for cancer treatment. Mater. Sci. Eng. C. Mater. Biol. Appl..

[23] Fattahpour S., Shamanian M., Tavakoli N., Fathi M., Sheykhi S.R., Fattahpour S. (2015). Design and optimization of alginate-chitosan-pluronic nanoparticles as a novel meloxicam drug delivery system. J. Appl. Polym. Sci..

[24] Abdel-Hafez S.M., Hathout R.M., Sammour O.A. (2018). Tracking the transdermal penetration pathways of optimized curcumin-loaded chitosan nanoparticles via confocal laser scanning microscopy. Int. J. Biol. Macromol..

[25] Lertsutthiwong P., Noomun K., Jongaroonngamsang N., Rojsitthisak P., Nimmannit U. (2008). Preparation of alginate nanocapsules containing turmeric oil. Carbohydr. Polym..

[26] Lertsutthiwong P., Rojsitthisak P., Nimmannit U. (2009). Preparation of turmeric oil-loaded chitosan-alginate biopolymeric nanocapsules. Mater. Sci. Eng. C..

[27] Chen D., Zheng H., Huang Z., Lin H., Ke Z., Xie S., Li B. (2012). Light-emitting diode-based illumination system for in vitro photodynamic therapy. Int. J. Photoenergy.

[28] Friedman A.J., Phan J., Schairer D.O., Champer J., Qin M., Pirouz A., Blecher-Paz K., Oren A., Liu P.T., Modlin R.L. (2013). Antimicrobial and anti-inflammatory activity of chitosan-alginate nanoparticles: A targeted therapy for cutaneous pathogens. J. Invest. Dermatol..

[29] Chopra D., Ray L., Dwivedi A., Tiwari S.K., Singh J., Singh K.P., Kushwaha H.N., Jahan S., Pandey A., Gupta S.K. (2016). Photoprotective efficiency of PLGA-curcumin nanoparticles versus curcumin through the involvement of ERK/AKT pathway under ambient UV-R exposure in HaCaT cell line. Biomaterials..

